# New molecules controlling endothelial barrier

**DOI:** 10.1186/cc14314

**Published:** 2015-03-16

**Authors:** S Jalkanen

**Affiliations:** 1 University of Turku, Finland

## Introduction

Acute lung injury (ALI) and its hypoxemic form, acute respiratory distress syndrome (ARDS), has no approved pharma cological treatment and is a condition with high mortality. Vascular leakage is one of the early events of ALI/ARDS. CD73 activity (ecto5'-nucleotidase) maintains the endothelial barrier function of lung capillaries via its enzymatic endproduct adenosine. Interferon-beta (IFNβ) increases synthesis of CD73 and has been effective in a mouse model of ALI [1].

## Methods

Therefore we conducted a phase I/II trial [2], in which intravenously administered human recombinant IFNβ1a (FP-1201) was used in the study, which consisted of dose escalation (phase I) and expansion (phase II) parts to test the FP-1201 safety, tolerability and efficacy in ALI/ARDS patients. CD73, MxA (a marker for IFN β response) and other biomarkers were measured to follow pharmacokinetics/ dynamics of the intravenously administered FP-1201 and therapeutic efficacy.

## Results

The optimal tolerated dose of FP-1201 (10 μg/day for 6 days) resulted in maximal MxA stimulation. Also soluble CD73 values increased, while IL-6 decreased in sera of the FP-1201-treated ALI/ARDS patients. The overall mortality of the 37 patients treated with FP-1201 was only 8.1%, fourfold to fivefold less than the expected rate based on APACHE II score values of 21.9. The control group (*n *= 59), which was eligible for the trial but not possible to recruit, had mortality of 32.2% (*P *= 0.01) and APACHE II score 23.0.

## Conclusion

Restriction of vascular leakage with FP-1201 seems to significantly benefit ALI/ARDS patients. Our results suggest that FP1201 could be the first effective, mechanistically targeted, disease-specific pharmacotherapy for patients with ARDS. However, these findings warrant conduction of a large multicenter study to establish safe and effective FP-1201 treatment of ARDS.
